# Analysis of Biotinidase Activity in Serum by Digital Imaging Colorimetry Detection

**DOI:** 10.1021/acsomega.3c05759

**Published:** 2023-10-13

**Authors:** Orhan Destanoğlu, M. Şerif Cansever, Esra İşat, Tanyel Zübarioğlu, A. Çiğdem Aktuğlu Zeybek, Ertuğrul Kıykım

**Affiliations:** † Institute of Forensic Sciences and Legal Medicine, Department of Science, Istanbul University-Cerrahpasa, Istanbul 34500, Turkey; ‡ Vocational School of Health Services, Department of Medical Services and Techniques, Istanbul University-Cerrahpasa, Istanbul 34265, Turkey; # Cerrahpasa Medical Faculty, Division of Nutrition and Metabolism, Department of Pediatrics, Istanbul University-Cerrahpasa, Istanbul 34098, Turkey

## Abstract

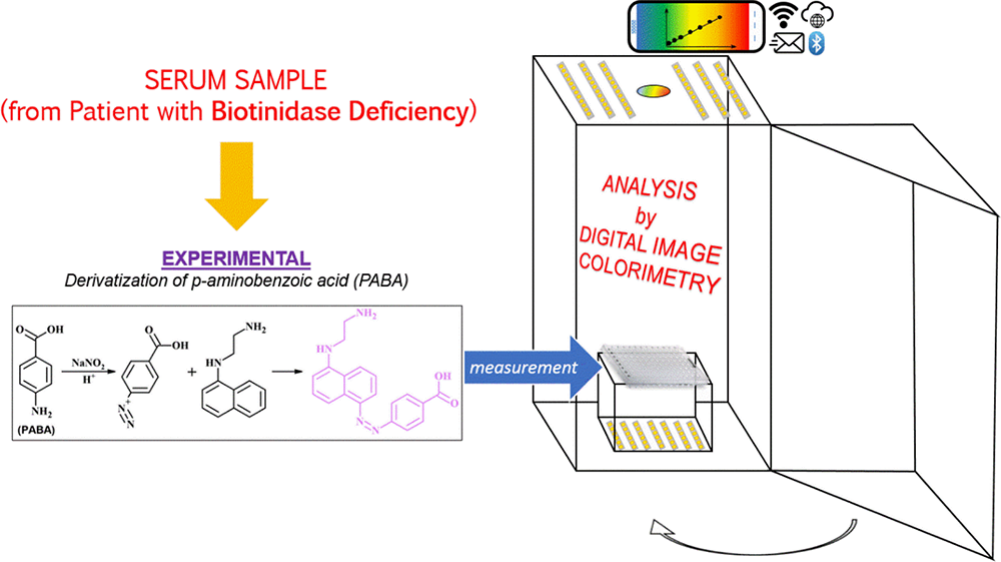

Biotinidase deficiency (BD) is an autosomal recessive inherited disorder of biotin recycling that leads to neurological and cutaneous consequences if left untreated. The clinical features of BD can be ameliorated or prevented by the administration of pharmacological doses of the vitamin biotin. Since it is a treatable disorder, BD is included in the newborn screening program in Türkiye as in many other countries. Therefore, monitoring of biotinidase enzyme activity (BEA) is of vital importance, especially for patients. The aim of this study was to develop a simple and reliable colorimetric method based on digital imaging for the analysis of BEA in serum samples. To determine the optimum distance and LED light source in the analyzer box that we fabricated in the laboratory, images of the solutions in a 96-well microplate were taken with a mobile phone camera, and each color space was examined. The most reliable relationship was between blank subtracted intensities of green channel and analyte concentrations, which was in the range of 35–400 ng/mL *p*-aminobenzoic acid (*r*^2^ = 0.999). The limit of detection and limit of quantification were 11 and 35 ng/mL, respectively. The proposed method was successfully applied to serum samples of 60 patients with BD and 60 healthy controls. We claim that the method can be easily performed for determination of BEA anywhere without needing expensive instruments.

## 1 Introduction

Biotin is an essential water-soluble vitamin and a cofactor of carboxylase enzymes. Biotinidase deficiency is an autosomal recessive inherited metabolic disorder that is caused by the biotinidase enzyme (EC 3.5.2.12) deficiency in the release and recycling of endogenous biotin from biocytin and short biotinyl peptides. In this context, progressive biotin depletion in biotinidase deficiency results in some complications such as biotinidase deficiency, deterioration in amino acid catabolism, fatty acid synthesis, and gluconeogenesis secondary to the effect on carboxylase reactions in where biotin acts as a cofactor.^1,2^ Patients with a residual enzymatic activity less than 10% are classified as “severe biotinidase deficiency”, and patients with activity levels between 10 and 30% are classified as “partial biotinidase deficiency”.^2^ Clinical severity of the disease is mainly dependent on the amount of dietary free biotin and residual enzyme activity. Untreated patients may develop resistance to seizures, hypotonia, lethargy, ataxia, developmental delay, hearing loss, optic atrophy, skin eruptions, and alopecia, although the clinical picture, which includes neurological and cutaneous symptoms and can progress to a comatose picture and lead to death, is often defined in severe biotinidase deficiency. In cases with partial deficiency, myopathy, peripheral neuropathy, and neuromyelitis optica may present with advanced age.^3^ Starting biotin treatment at a dose of 5–20 mg/day, depending on the level of the underlying enzymatic insufficiency, is effective in preventing the occurrence of clinical findings, and treatment initiated after clinical findings develop is effective in improving the symptoms.^1^

The incidence of biotinidase deficiency varies between 1:40,000–60,000 live births. It is known that the incidence of the disease, which is widely included in newborn screening all over the world, varies regionally, and its incidence increases up to 1:9,000.^4^ The incidence of biotinidase deficiency in Türkiye, which has started to be screened within the scope of the “National Newborn Screening Program” in our country since 2008, has been reported as 1:7,116 in the literature.^5,6^ Istanbul University-Cerrahpasa, Cerrahpasa Medical Faculty, Department of Pediatrics, Division of Nutrition and Metabolism and Laboratory, where this study was conducted, is one of the leading centers in Türkiye. It has 40 years of experience in the diagnosis, treatment, and follow-up of patients with biotinidase deficiency. Due to the high incidence of biotinidase deficiency in Türkiye, many patients and analysis of biotinidase levels are referred to our department. In addition, our department of inborn errors of metabolism has participated in the national newborn screening program biotinidase deficiency from the beginning.

Although there are different methods such as radioassays,^7^ fluorometric assays,^8,9^ genetic analyses,^10−12^ and digital microfluidic enzyme assays^13^ for the determination and diagnosis of biotinidase enzyme activity, the most commonly used method is the colorimetric measurement^14−16^ of the enzyme activity in plasma or serum samples. The mean normal biotinidase activity in healthy people has been reported as 4.4–10 nmol/min/mL and the standard deviation as 7.1 ± 1.2 nmol/min/mL^2^. In our country, biotinidase activity is determined in dry blood samples taken within the scope of national newborn screening. With the effective implementation of the screening program, cases were diagnosed at an early stage, and life without sequelae became possible.

Colorimetric methods used to determine the concentration of target analytes are widely used in biochemistry laboratories by comparing or measuring the color depth resulting from the chromogenic reaction of colored compounds.^17^ In routine laboratories, the most commonly used method for determination of biotinidase activity in serum is based on colorimetric measurement of *p*-aminobenzoate (PABA) released from one of the analogs of biocytin, namely, *N*-biotinyl-*p*-aminobenzoate (Biotin-PABA).^1,2,14,18^ On the other hand, in recent years, numerous scientific studies have been interested in the use of digital imaging (DI) for monitoring the colorimetric reactions. DI colorimetry (DIC), which has become a popular research topic in recent years, is an advanced colorimetric method for measuring target analyte after digitizing the colors in the captured images by processing software. Smartphones, scanners, webcams, and the other digital cameras can be used as optical sensors to collect images for measuring light intensities transmitted from a sample.^17,19^ With the availability of these low-cost imaging tools, the possible risk for accessing conventional colorimetric analyzers has reduced. The color models or spaces such as RGB (red, green, blue), HSB (hue, saturation, brightness), and CMYK (cyan, magenta, yellow, black) can be useful for employing the DI devices as low-cost colorimetric detectors. Therefore, the channels of the color spaces can be correlated with concentrations of the target analytes for quantification.^19,20^ Considering the activities that can be done in different ways such as social media monitoring, banking transactions, business transactions, games, and how we monitor our health via smartphones, these devices have inevitably permeated almost every aspect of our lives. Essentially, the health status of people can be monitored by detecting small molecules in biological materials with a smartphone. In the literature, many recent studies have utilized the DIC methods for determination of various analytes such as metals/heavy metals, herbicides, pesticides, antibiotics, biological and medical markers, natural compounds, and bacteria/viruses in different types of matrices.^19−28^

This study aimed at developing a simple, accurate, sensitive, and reliable DIC-based method as an alternative to the routine method^18^ employing a UV spectrometer for analysis of biotinidase enzyme activity in serum samples. To the best of the authors’ knowledge, no study has yet been focused on a DIC method for analysis of biotinidase activity yet. Therefore, after the comprehensive optimization studies had been conducted, 120 real sample solutions were measured with both a routinely employed UV spectrometer and the proposed DIC system. Statistical analyses revealed that a reliable DIC method for the determination of biotinidase activity was accomplished. It is noteworthy that the developed DIC method was superior to UV spectrometers in terms of multiple standard and sample solutions in a 96-well micro test plate could be simultaneously measured in a single image taken by a smartphone camera. Besides, the proposed DIC method may be used not only in routine laboratories but also in some health clinics and outside traditional laboratories as a point-of-care (POC) test because it is fast, easy-to-use, and reliable. Considering that almost everyone uses a smartphone with a camera today, the proposed method has great potential to become widespread in practice.

## 2 Materials and Methods

### 2.1 Reagents

Trichloroacetic acid (TCAA), ammonium sulfamate (AMS), *N*-1-naphthyl-ethylenediamine (NED), disodium ethylenediaminetetraacetate dehydrate (Na_2_EDTA·2H_2_O), human albumin, PABA, and biotin-PABA were purchased from Sigma (St. Louis, USA). NaNO_2_ and K_2_HPO_4_·3H_2_O were obtained from Merck (Darmstant, Germany). KH_2_PO_4_ was obtained from Riedel-De-Haën (Hannover, Germany).

Ultrapure deionized water (resistivity ≥ 18.2 MΩ.cm) used for preparing the solutions was obtained from a Milli-Q water system (Merck Millipore, Billerica, MA, USA).

A sterile, transparent, flat base, and polystyrene 96-well micro test plate was purchased from SARSTEDT AG & Co. KG (Nümbrecht, Germany).

### 2.2 Solutions

A 30% (v/v) aqueous TCAA solution was prepared by diluting 3 mL of pure TCAA with deionized water in a 10 mL volumetric flask.

To prepare the solutions of NaNO_2_ and NED with a concentration of 0.01% (w/v), 10 mg of solid chemicals was weighed from their pure stocks and diluted to 10 mL with deionized water in different volumetric flasks.

To prepare 0.05% (w/v) of AMS solution, 50 mg of solid AMS was weighed, and after it was dissolved in some water, the solution was diluted with deionized water in a 10 mL volumetric flask.

Preparation of the Buffer A solution at pH = 6 was as follows: after weighing 0.91 g of KH_2_PO_4_, 1.52 g of K_2_HPO_4_·3H_2_O, 0.092 g of EDTA, 26.3 mg of human albumin, and 6 mg of biotin-PABA, the mixture was dissolved and diluted with deionized water in a 100 mL volumetric flask.

Preparation of the Buffer B solution at pH = 6 was as follows: after weighing 0.91 g of KH_2_PO_4_, 1.16 g of K_2_HPO_4_·3H_2_O, 0.092 g of EDTA, and 26.3 mg of human albumin, the mixture was dissolved and diluted with deionized water in a 100 mL volumetric flask.

A total of 34.3 mg of PABA was weighed, and after dissolving, it was diluted with deionized water in a 100 mL volumetric flask to prepare 2.5 mM of its stock standard solution. Calibration solutions of PABA were prepared by diluting appropriate proportions of the stock standard solution with deionized water.

### 2.3 Principle of Determination of Free PABA

**Figure 1:**
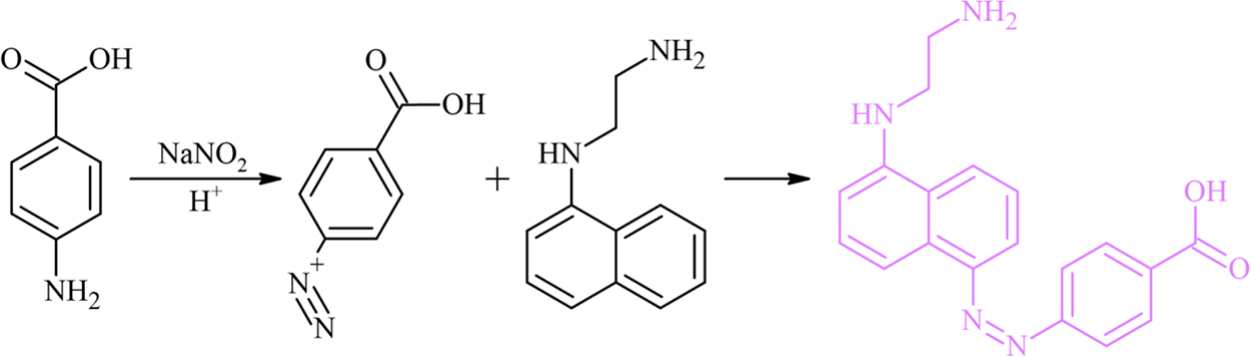
Derivatization reaction of PABA to the pink/purple diazo dye.

In our laboratory, a very common spectrophotometric method is used for routine analysis of biotidinase enzyme activity.^14^ In principle, to analyze free PABA by using a colorimetric method in the visible region of the electromagnetic spectrum, a pink/purple colored solution that was formed after derivatizing PABA on the basis of the Griess reaction^29,30^ was measured. In the first step, PABA reacted with NaNO_2_ to form its diazonium salt. After the excess concentration of NaNO_2_ was removed by adding AMS, diazonium salt of PABA reacted with NED used as a coupling agent to yield the diazo dye, giving pink/purple color. The reaction is exhibited in Figure 1. This mechanism is the base for determination of PABA concentration and accordingly the biotinidase enzyme activity. In this study, the pinkish colored solution was measured both by the standard method applied in routine analyses at 546 nm by using a spectrophotometer and the proposed DIC method under the optimized conditions.

### 2.4 Sample Collection and Storage

A 2–3 mL serum sample from blood was obtained by collecting blood into Vacutainer tubes with no additive by venipuncture. In order not to decrease the serum biotidinase enzyme activity, the blood samples taken into the tube with a serum separator were delivered to the laboratory. After the whole blood sample was immediately centrifuged (<1 h from collection) to separate the cells, the serum sample was frozen at −80 °C to prevent enzyme activity loss until analysis. Enzyme activity is stable in serum at −20 and −80 °C for more than 2 and 5 months, respectively. However, significant activity loss can occur if the sample is kept at higher temperatures (+4 °C or room temperature).^16,31^

The study protocol was designed according to the principles of Ethical Guidelines for Biomedical Research Involving Human Subjects as defined in the Declaration of Helsinki and was approved by the Ethical Committee of Istanbul University-Cerrahpaşa, Cerrahpaşa Medical Faculty. All parents of the patients included in the present study gave informed consent.

### 2.5 Sample Preparation for Colorimetric Analysis

The sample preparation procedure for the proposed DIC method was utilized the same as the common procedure^14^ routinely conducted also in our laboratory prior to the colorimetric determination of PABA using a spectrophotometer. A Secomam, S.750, (France) spectrophotometer was utilized to perform the standard method^14^ for comparison with the proposed DIC method for determination of PABA. The colorimetric method is based on the measurement of the pinkish azo-dye product formed by coupling reaction between NED and free PABA liberated from biotin-PABA that is an artificial substrate when reacting with enzyme biotinidase.^2,14−16,18^

A 950 μL Buffer A solution and 50 μL serum sample were placed in a test tube, and then it was vortexed. After this solution was kept at 37 °C for 30 min for incubation with the substrate BAA yielding PABA, the enzymatic reaction was stopped by adding 100 μL of 30% (v/v) TCAA. The tube was centrifuged at 10,000 rpm for 5 min. Then, to diazotize the PABA released as a result of the biotinidase enzyme activity in the serum during the reaction, 500 μL of the supernatant was mixed with 250 μL of deionized water and 100 μL of 0.01% (w/v) NaNO_2_ in another clean tube, and it was kept for 3 min. To remove excess nitrite, 100 μL of 0.05% (w/v) ammonium sulfamate was added in the solution, and it was incubated for 3 min. Consequently, to obtain pink/purple colored solution, 100 μL of 0.01% (w/v) NED was pipetted in the solution, vortexed, and waited for 10 min for completion of the reaction with PABA.^15,16^

### 2.6 DIC System and Data Acquisition

The digital images were taken by using a smartphone Huawei Mate 20 lite ((SNE-LX1), Shenzhen, China) with 20 Mpixels + 2 Mpixels dual rear cameras (f/1.8 aperture, 27 mm wide, phase detect autofocus (PDAF), 5120 × 3840 pixels resolution). The flash light of the smartphone and high dynamic range (HDR) were deactivated. The images were saved in JPEG format with a size of 3 MB in the smartphone’s memory. After experiencing a number of applications, the “RGB Color Detector” app, which is available on the Google Play Store^32^ or from Apple Store,^33^ was chosen as the most useful one for our study since (I) photographs could be processed by either using the phone’s camera directly or loading from gallery, (II) RGB, CMYK, HSV, HEX, and HSL values could be exported online in CSV format for processing by computer software, but also the most useful option that we used was “Analyze color for chemical assay” allowing determination of the concentration of PABA after calculating linear regression data (slope, intercept, and correlation coefficient) from the appropriate color formats of standard measurements.

**Figure 2:**
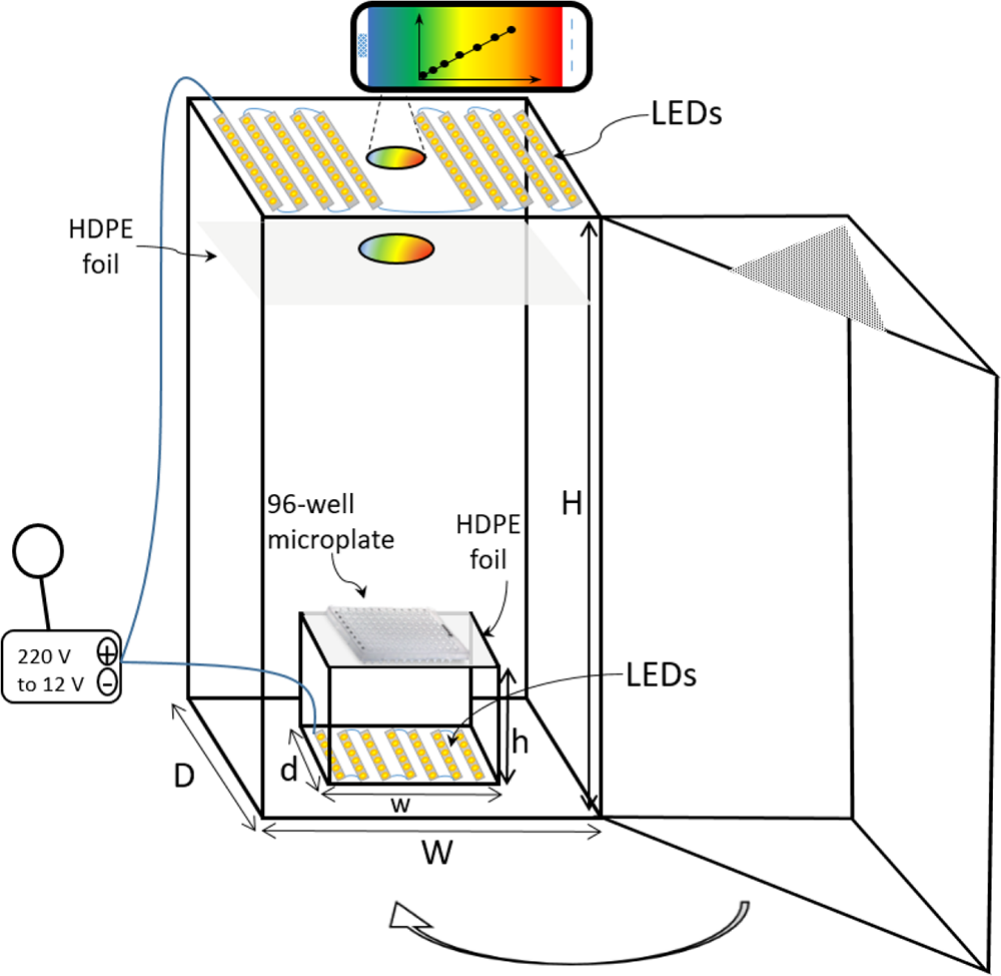
Schematic illustration of the custom-built system employed for taking digital images by the camera of a smartphone (*H* = 21 cm, *W* = 15 cm, *D* = 12 cm; *h* = 4 cm, *w* = 11 cm, *d* = 10 cm).

In this study, we fabricated an analyzer box made of cardboard with dimensions of 21 × 15 × 12 cm (height × with × depth) that was employed for acquisitions (see Figure 2). The camera of the smartphone was placed to the centered fitting hole on the top of the box, which was positioned vertically 16 cm right above the 96-well microplate. To ensure that the photo shoots were taken from the same point, a black silicone phone case was fixed by attaching it to the hole of the box in a suitable position with silicone adhesive. With the use of a fixed phone case, possible stray light interferences that may come between the camera and the box were also eliminated. The uplight and downlight illuminations were provided by 12 V yellow LED strips attached both to the upper part of the inner box and in the bottom of the open-top clear white plastic small box (4 × 11 × 10 cm) with the lengths of 100 and 70 cm, respectively. To disperse the light from the LEDs, a recycled (resin ID code: 2) white plastic foil, its chemical composition being high-density polyethylene (HDPE), was used after cutting appropriate dimensions. The sheets were taped to 3 cm in front of uplight and top of the small box on where the 96-well microplate was placed. Since the triangular prism-shaped cover wrapped the box when it was closed, any interference was prevented from the environment light. Additionally, the outer edges of the box were covered with a black tape just in case. Measurements were conducted by placing 96-well microplates on top of the downlight source box. After trying different function combinations of the channels in the color spaces, the G color channel was utilized to obtain the analytical responses of the photographs. All statistical calculations were made by using MS Office Excel 2016 software after exporting the data in CSV format from the application as the results were displayed one by one in the smartphone.

On the other hand, the disposable UV cuvettes containing the standard and sample solutions were photographed by turning our custom-built box at an angle of 90° immediately after their measurements were completed in the spectrophotometer. However, in our study, the use of a 96-well plate was preferred because it was more advantageous in that only up to 10 UV cuvettes could be used in a single measurement and their sequencing was more difficult.

## 3 Results and Discussion

The proposed DIC method was developed for determination of biotinidase enzyme activity in serum samples by using a smartphone as an alternative to the widespread routinely used colorimetric method based on measuring the concentration of PABA.^1,14,18^ The sample preparation procedure was conducted the same as that of the standard colorimetric method until the measurement.^14^ Thereafter, the images were captured from the solutions that were separately put in the cuvettes of the UV/vis spectrometer and in a 96-well microplate in the custom-built analyzer box under different conditions. The optimization studies for the LED types and the distances between the camera and solutions were carried out comprehensively to determine the relationships between the concentration of PABA and intensities of the color channels.

The RGB color space generally used in displays is the combination of different intensities of R, G, and B ranging from 0 to 255 for each channel. For instance, RGB values of black, white, magenta, purple, and blue are (0,0,0), (255,255,255), (255,0,255), (128,0,128), and (0,0,255), respectively.^20^ The CMYK model, which is the subtractive model utilized for color printing generally, is a variation of CMY and blacK. The reason for adding K is to improve the quality of dark colors. CMYK ranges are between 0 and 100% for the channels.^21^ The data of the channels of the color space modes (RGB, CMYK, HSV, and HSL) of the images collected from different distances and LED light sources were exported to find out the optimal relationships between the response and the analyte concentration. Therefore, the subtracted intensities (*I* – *I*_blank_), absorbances (*A* = log(*I*_blank_/*I*)), and average values of the channels were evaluated one-by-one. Since a single result value could be obtained from the mobile application, standard deviation and other statistical calculations were done in MS Excel after exporting the measured values. We initially obtained totally 320 graphs, and then, 56 graphs were primarily chosen because their calibration correlation coefficients were ≥0.995 at least four concentration points. Then, determination of the most accurate results was tried by calculating the error% values between the DIC results and the spectrophotometer results of four real samples.

### 3.1 Determination of Optimal Analytical Response for 96-Well Microplate Application

**Figure 3:**
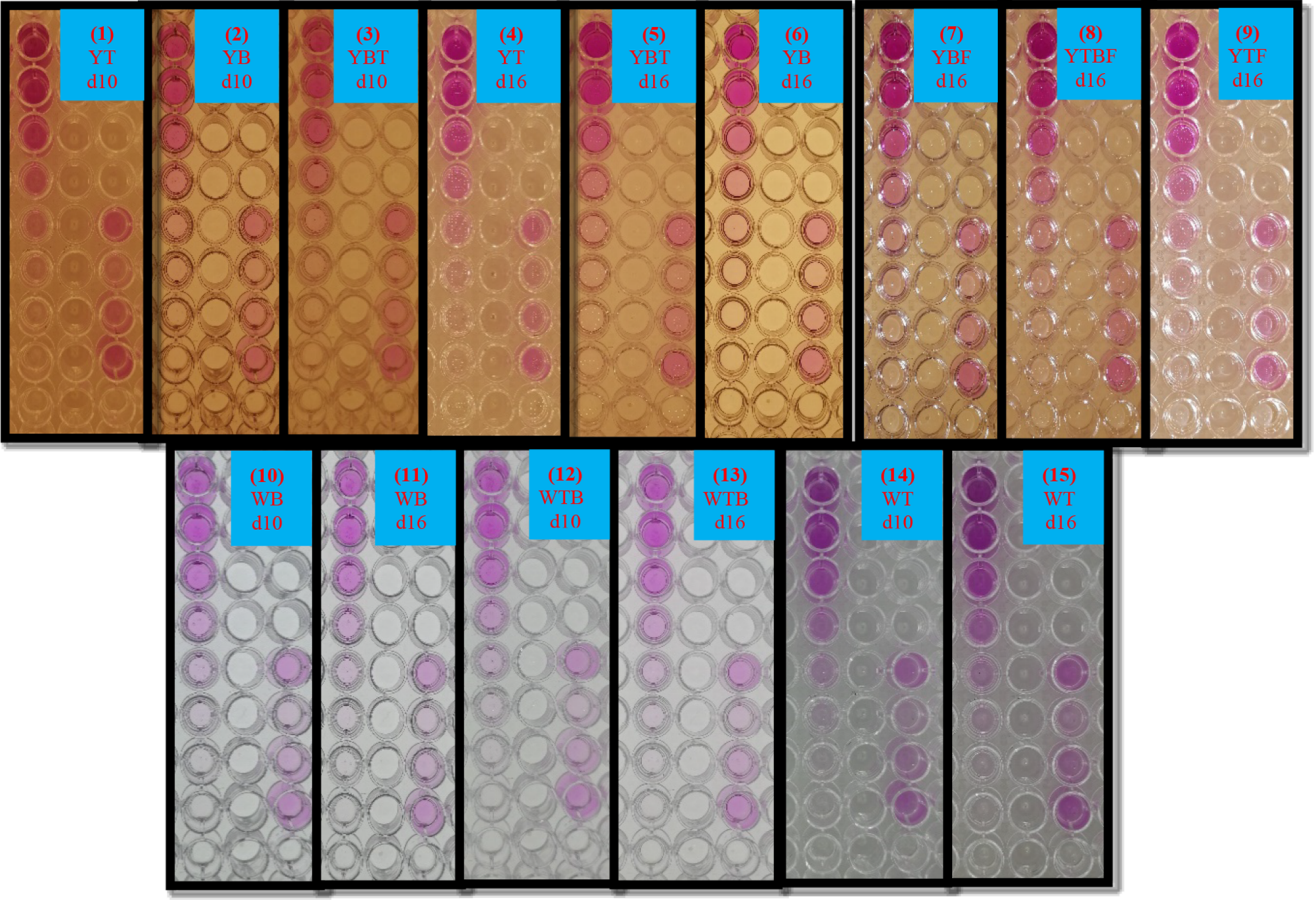
Images taken in different conditions of the solutions in a 96-well plate. Left solutions: PABA standards, right solutions: four real samples. Letter codes of the conditions; Y: yellow LED, W: white LED, B: bottom, T: top, d10: 10 cm distance, d16: 16 cm distance.

First, a series of standard solutions of PABA with concentrations of 20, 40, 80, 160, 320, 640, 1250, and 2500 nmol/mL were prepared. In the studies where measurements were conducted using a 96-well microplate, optimizations were started after 250 μL of blank, standard, and sample solutions were pipetted. Four samples with different concentrations, which were previously measured by spectrophotometer at 546 nm, of PABA were used. In the optimization study, the images were taken at 10 and 16 cm distances between the camera and solutions. The white and yellow LED light sources (only bottom, only top, bottom + top) were separately turned on for each distance. On the other hand, the photographs were also taken using the smartphone flash when the yellow LED light was on. However, use of flash was abandoned as it affected the image since crescent-shaped light refractions and shadows occur (see Figure 3-(7), (8), and (9)), especially when the upper LED light source was not switched on.

**Figure 4:**
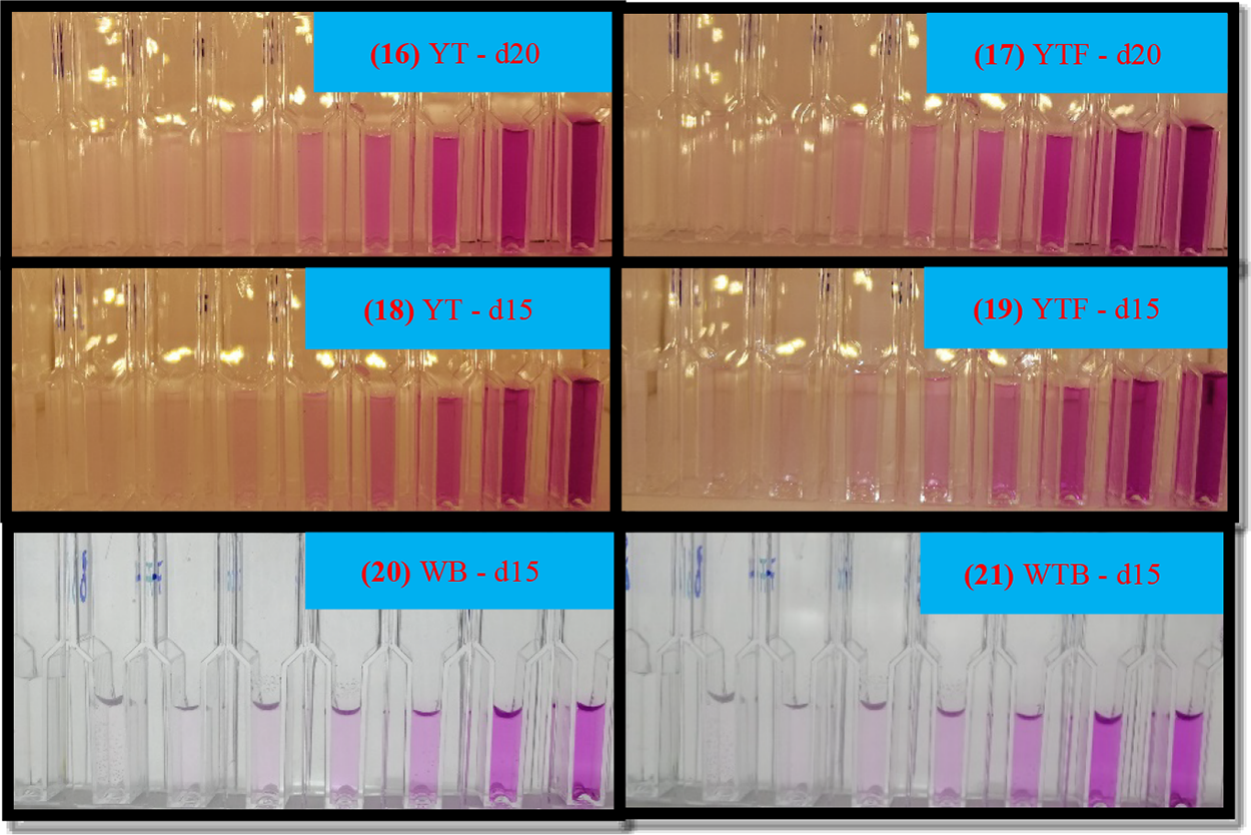
Images taken in different conditions of the standard solutions presented in cuvettes.

As seen in Figure 3-(1) and (4), the reflections were observed at some points in the solution if the upper LED lights were not dispersed. A similar image was observed when the phone flash was turned on. In addition to causing difficulties in selecting the area to collect data from the images, these reflections naturally affected the accuracy and reproducibility of the results since they actually gave rise to significant differences in the values of the color channels. For this reason, to prevent these fluctuations in the results of the measurements, a piece of HDPE blown film with the edges and middle cut in appropriate sizes was glued 3 cm below the top light source, and thus, the light was dispersed.

Then, among all of the measurements in the microplates, eight of the results with good error% values were selected (see Table 1). The lowest concentrations differed between 20 and 80 nmol/mL while the highest concentration was found to be 640 nmol/mL for all in the linear ranges. The best relationships could be established when the linear curves were constructed between blank subtracted intensities (*I* – *I*_blank_) of the G, M, and S channels and analyte concentrations in 96-well microplate examinations.

**Table 1:** Summary of the Selected Conditions (*r*^2^ > 0.997), Results of the Biotinidase Enzyme Activities of Four Real Samples, and Their Error% Values Obtained from the Measurements of the Solutions in the 96-Well Microplate

				linear range						result (U/L)		
no.	color space	channel	relationship	PABA μmol/L	as-activity (μmol/L/min)	correlation coefficient	slope	intercept	sample no.	DIC	Std method	bias%
(5)	RGB	G	*I* – *I*_blank_	40–640	1.3–21.3	0.9997	–0.1836	5.6078	S1	7.8	7.6	2.4
									S2	2.6	2.7	–3.9
									S3	5.1	5.0	2.3
									S4	9.3	8.7	6.4
(9)	CMYK	M	*I* – *I*_blank_	80–640	2.7–21.3	0.9975	3.5057	2.422	S1	8.0	7.6	5.2
									S2	3.1	2.7	13.2
									S3	4.9	5.0	–1.3
									S4	8.2	8.7	–5.3
(10)	CMYK	M	*I* – *I*_blank_	40–640	1.3–21.3	0.9991	2.5431	0.7611	S1	8.5	7.6	11.7
									S2	2.6	2.7	–2.0
									S3	5.2	5	4.6
									S4	9.6	8.7	10.1
	HSV	S	*I* – *I*_blank_	40–640	1.3–21.3	0.9986	2.5721	–2.2148	S1	8.5	7.6	11.8
									S2	2.7	2.7	1.0
									S3	5.3	5	5.6
									S4	9.6	8.7	10.1
(11)	CMYK	M	*I* – *I*_blank_	40–640	1.3–21.3	0.9991	2.4607	–0.7601	S1	8.3	7.6	8.9
									S2	3.1	2.7	14.5
									S3	5.0	5	–0.5
									S4	9.3	8.7	6.7
	HSV	S	*I* – *I*_blank_	20–640	0.7–21.3	0.9991	2.44	–2.792	S1	8.3	7.6	8.7
									S2	3.0	2.7	12.5
									S3	4.9	5	–1.3
									S4	9.3	8.7	6.7
(12)	CMYK	M	*I* – *I*_blank_	40–640	1.3–21.3	0.9986	2.8277	–1.8319	S1	8.4	7.6	9.9
									S2	3.1	2.7	13.5
									S3	5.3	5.0	6.5
									S4	9.6	8.7	10.5
	HSV	S	*I* – *I*_blank_	20–640	0.7–21.3	0.9973	2.7996	–4.549	S1	8.3	7.6	9.7
									S2	3.0	2.7	11.0
									S3	5.3	5.0	5.6
									S4	9.6	8.7	10.5

According to Table 1, although all correlation coefficients of the selected eight studies were above 0.997, the best error% values for four different concentrations of the real samples were obtained when the “*I*_G_ – *I*_Gblank_” from condition (5) was utilized. Consequently, the optimal condition was found as the best analytical responses for determination of PABA and accordingly of biotinidase enzyme activity.

As seen in Table 1, according to the results of trial no. 10, where the white LED light source was applied from a distance of 16 cm from below, successful results were found for low concentrations with subtracted intensities of “*I*_M_ – *I*_Mblank_” and “*I*_S_ – *I*_Sblank_”, while errors increased at concentrations above 150 nmol/mL. As a result of the study no. 11 carried out in the M and S channels at a distance of 16 cm to the top-white LED light, the acceptable error was found only about the middle concentration level (150 nmol/mL PABA), while the error values were found to be higher at the low and high concentration levels.

The error% found in the results of the M and S channels was approximately in the range of 5–13% in experiment no. 12, where the white light was applied at a distance of 10 cm from the lower and upper light source (Table 1). On the other hand, the sensitivity was found weak in experiment no. 9 in which the upper yellow LED light and the smartphone flash were turned on during capturing. Accordingly, the error% value of the lower concentration sample (S2) measurement was naturally found to be 13.2%. However, the M channel was successful for measurements of the other samples under the same conditions.

### 3.2 Determination of Optimal Analytical Response in Cuvettes

While the images of the solutions in the microplate were captured by the smartphone’s camera vertically, the images were taken for the solutions in the cuvette by turning the analyzer box horizontally. The images captured from the cuvettes are given in Figure 4.

After evaluating all the results, four of them (nos. 18 and 20) with better error% values were chosen. The results are summarized in Table 2. Better relationships were obtained not only with the blank subtracted intensities of G and M channels but also with log(*I*_b_/*I*) of the G channel and average values of RGB channels. Even though all correlation coefficients of the selected conditions were above 0.997, the best error% values (1–6%) for the real samples were obtained from the condition 18 (log(*I*_Gb_/*I*_G_)), which was found to be the optimal condition for determination of PABA in serum samples in the concentration range of 20–640 nmol/mL.

**Table 2:** Summary of the Selected Conditions (*r*^2^ > 0.997), Results of the Biotinidase Enzyme Activities of Four Real Samples, and Their Error% Values Obtained from the Measurements of the Solutions in the Cuvettes

				linear range						result (U/L)		
no.	color space	channel	relationship	μmol/L	as-activity (μmol/L/min)	correlation coefficient	slope	intercept	sample no.	DIC	Std method	bias%
(18)	RGB	RGB/3	log(Ib/*I*)	20–640	0.7–21.3	0.9975	0.0165	–0.0115	S1	8.3	7.6	9.6
									S2	3.3	2.7	23.4
									S3	5.2	5.0	3.3
									S4	9.1	8.7	4.5
	RGB	G	log(Ib/*I*)	20–640	0.7–21.3	0.9993	0.0449	0.0064	S1	8.1	7.6	6.1
									S2	2.8	2.7	4.3
									S3	5.1	5.0	1.0
									S4	9.0	8.7	3.0
(20)	RGB	G	*I* – *I*_blank_	20–640	0.7–21.3	0.9991	–6.6242	2.5628	S1	7.4	7.6	–3.0
									S2	3.1	2.7	13.1
									S3	4.8	5.0	–3.8
									S4	8.6	8.7	–0.9
	CMYK	M	*I* – *I*_blank_	40–640	1.3–21.3	0.9978	3.365	1.2996	S1	6.9	7.6	–9.0
									S2	2.4	2.7	–11.2
									S3	4.5	5.0	–10.4
									S4	8.6	8.7	–0.7

Among the other three results given in Table 2, acceptable results could be achieved for S1, S3, and S4 where the relationship between the subtracted intensity of G channel (*I*_G_ – *I*_Gblank_) and concentration was established in the experiment no. 20 except for S2 with a higher bias% (13%) than the others (<4%). We, therefore, concluded that this condition was not suitable for low and around threshold concentration values.

Consequently, we found out two different optimal conditions for determination of PABA concentration in the serum solutions using a 96-well microplate and the UV cuvette placed in our custom-built analyzer box. However, although reliable results were obtained in the first trials, the cuvettes were not used for the analysis of real samples after this point since more measurements could not be conducted at once and it is more difficult in terms of practicality of installation compared to the microplate. Therefore, measurements of the real samples were continued by utilizing only the 96-well microplates.

### 3.3 Analytical Figures of Merit

**Figure 5:**
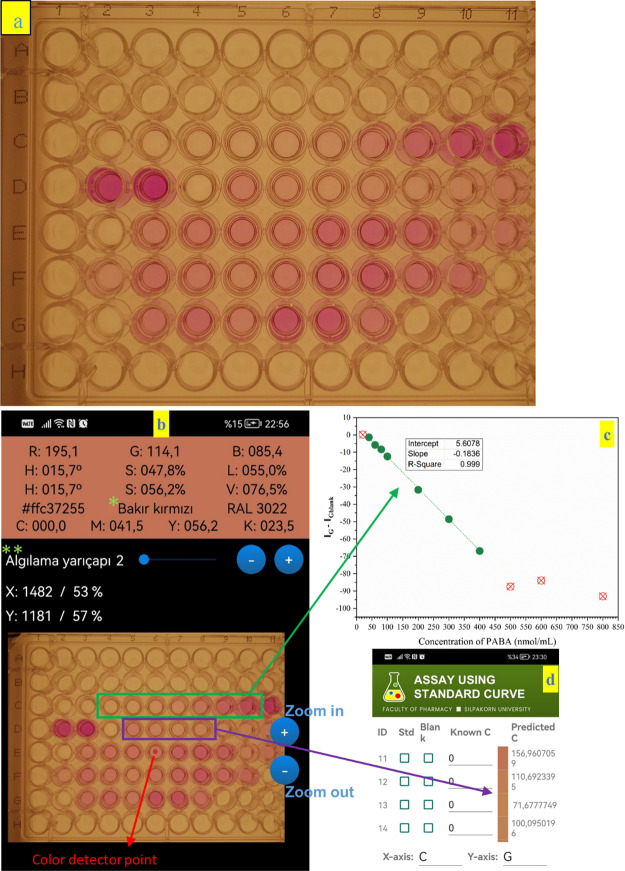
(a) Image of a 96-well microplate containing the standard and sample solutions for DIC analysis. The positions of the solutions were as follows: C2: blank; C3–C10: calibration solutions (35–400 nmol/mL); C11–D3: 500, 600, and 800 nmol/mL standard PABA (above the linear range); D4, E2, F11, G2: blank; D5–G8: real sample. (b) Screenshot revealing the color channel measurements on a 96-well microplate by a mobile app (*copper red, **detection radius). (c) Linear calibration curve. (d) Screenshot showing PABA concentration results in the samples D5–D8 measured with the mobile app.

Biotinidase enzyme activity determination was carried out using the 96-well microplate due to its advantages such as measuring tens of standard or sample solutions at one time, easy setup, and requiring less volume of the solutions. In optimization studies, in addition to linearity, the experimental conditions that would yield reliable results were determined by evaluating the results of four real samples with low and high concentrations. After the solutions were pipetted into a microplate, the photographs were taken from a distance of 16 cm when the top and bottom yellow LED light sources were turned-on in the analyzer box that we fabricated in the laboratory. The relationships between blank subtracted intensities of G channel (*I*_G_ – *I*_Gblank_) extracted from the images and analyte concentrations were assessed. The performance characteristics of the proposed DIC method were as follows: sensitivity, linearity, accuracy, and precision. For sensitivity, the limit of detection (LOD) and limit of quantification (LOQ) were calculated by multiplying the standard deviation obtained from the results of 10 blank solution measurements by 3 and 10, respectively. To determine the linear calibration range exactly, PABA solutions at concentrations of 20, 40, 60, 80, 100, 200, 300, 400, 500, 600, and 800 nmol/mL were prepared and 250 μL of each solution was pipetted into a 96-well microplate. Although the correlation coefficient of the calibration curve was found >0.999 up to 600 nmol/mL concentration, the upper quantification point of the calibration curve was assigned as 400 nmol/mL since the bias% error increased above 400 nmol/mL in the measurements carried out with DIC and standard methods on real samples. Calibration curves are shown in Figure 5c. Therefore, the samples with >400 nmol/mL PABA were adequately diluted for measuring in the linear range. The parameters of sensitivity and linearity are summarized in Table 3.

**Table 3:** LOD, LOQ, Linear Range, Regression Equation, and Correlation Coefficient Values of the Proposed DIC Method

LOD (ng/mL)	11
LOQ (ng/mL)	35
linear range (ng/mL)	35–400
regression equation	*y* = −0.1836*x* + 5.6078
correlation coefficient (*r*^2^)	0.999

In addition, to evaluate the accuracy and precision of the proposed DIC method, the mean and standard deviation (SD) values (*n* = 6) obtained from six real samples at low, medium, and high concentration levels were statistically compared with the standard method’s results (*n* = 6) by utilizing the Student’s *t-*test and *F*-test at 95% confidence level, respectively. According to statistical results presented in Table 4, there were no significant differences between the two methods’ mean values and SD values since both *t*_calculated_ and *F*_calculated_ values were found below their critical values, which were 2.23 and 5.05, respectively. It is noteworthy that an accurate and precise DIC method for determination of PABA in serum samples and accordingly of biotinidase enzyme activity was developed. Very good RSD% values were found (<6) except for the first sample as its concentration (37 nmol/mL) was close to the LOQ. In addition, 3 U/L is critical for the biotinidase enzyme activity value, which is equal to 100 nmol/mL PABA and very reliable results are obtained at this concentration level (see Table 4).

**Table 4:** *F*-Test and Student’s *t*-Test Results between the DIC Method and the Standard Method for Evaluating Precision and Accuracy of the Proposed DIC Method, Respectively

reference method		DIC			*F-*test		Student *t-*test	
mean ± SD (nmol/L)	RSD%	mean ± SD	RSD%	bias%	*F*_calc._	*F*_critical_ (*p* = 0.05)	*t*_calc._	*t*_critical_ (*p* = 0.05)
33 ± 5	16	37 ± 5	13	13	1.17	5.05	1.38	2.23
70 ± 3	5	67 ± 4	6	–4	1.53		1.27	
98 ± 3	4	96 ± 4	4	–2	1.40		0.81	
132 ± 3	2	132 ± 1	1	0	4.68		0.22	
209 ± 8	4	201 ± 6	3	–4	2.15		1.60	
327 ± 9	3	317 ± 8	3	–3	1.22		1.81	

### 3.4 Analysis of Real Serum Samples by the DIC Method

The serum samples taken from healthy people and from the patients suffering from biotinidase enzyme deficiency were prepared as described in the sample preparation section prior to analysis. Each sample was analyzed using both the proposed DIC method and the standard spectrophotometric method. Figure 5a demonstrates the image of a 96-well microplate containing the standards and 31 sample solutions that was placed in our custom-built DIC analyzer box just before processing. The screenshot showing the measurement of the color channels in the image using the mobile application is given in Figure 5b. No solution was added to any well in column 1 of the 96-well microplate. A blank solution was added to the well at position C2, and the standard PABA solutions with a concentration range of 20–800 nmol/mL were added to the following wells until D3. Measurements were conducted in the linear concentration range revealed in Figure 5c. The screenshot of the mobile app showing the results of real samples in the position range of D5–D8 is presented in Figure 5d. What is more, all results of 120 real samples are presented in Table 5 in order of increasing age of people. The results given in Figure 5d belong to samples nos. 10, 52, 1, and 17 in Table 5.

**Table 5:** PABA Concentration and Biotinidase Enzyme Activity Results of Real Serum Samples Measured by the Proposed DIC Method

no.	gender	year	month	PABA (nmol/mL)	RSD%	activity (U/L)
1	F	0	1	70 ± 3	5	2.33
2	F	0	1	84 ± 5	6	2.80
3	F	0	1	68 ± 5	7	2.27
4	F	0	1	178 ± 6	4	5.93
5	M	0	1	160 ± 3	2	5.33
6	M	0	1	161 ± 7	4	5.37
7	M	0	1	123 ± 6	5	4.10
8	M	0	1	140 ± 3	2	4.67
9	F	0	1	196 ± 5	2	6.53
10	F	0	1	150 ± 8	5	5.00
11	F	0	1	87 ± 5	6	2.90
12	F	0	1	84 ± 13	16	2.80
13	M	0	1	124 ± 3	1	4.13
14	M	0	1	82 ± 2	2	2.73
15	M	0	1	196 ± 7	4	6.53
16	F	0	2	82 ± 5	6	2.73
17	M	0	4	98 ± 3	4	3.27
18	M	0	4	119 ± 3	3	3.97
19	M	0	4	189 ± 1	2	6.30
20	M	0	5	241 ± 10	4	8.03
21	F	0	5	97 ± 5	5	3.23
22	M	0	5	176 ± 3	2	5.87
23	F	0	5	123 ± 3	2	4.10
24	M	0	5	ND	-	-
25	F	0	6	56 ± 6	11	1.87
26	M	0	6	99 ± 6	6	3.30
27	M	0	6	260 ± 7	3	8.67
28	F	0	6	216 ± 5	2	7.20
29	F	0	6	162 ± 6	3	5.40
30	M	0	6	77 ± 3	4	2.57
31	M	0	7	191 ± 2	1	6.37
32	M	0	7	171 ± 3	2	5.70
33	F	0	8	222 ± 5	2	7.40
34	F	0	8	83 ± 6	8	2.77
35	F	0	10	218 ± 5	2	7.27
36	F	0	11	198 ± 7	4	6.60
37	F	0	11	280 ± 5	2	9.33
38	M	1	3	94 ± 3	3	3.13
39	M	1	3	23 ± 5	20	0.77
40	M	1	5	287 ± 6	2	9.57
41	F	1	5	255 ± 5	2	8.50
42	M	1	8	132 ± 3	2	4.40
43	M	1	9	191 ± 7	4	6.37
44	F	1	11	191 ± 7	4	6.37
45	M	2	1	156 ± 5	3	5.20
46	F	2	2	361 ± 8	2	12.03
47	M	2	5	128 ± 6	5	4.27
48	F	2	6	142 ± 12	9	4.73
49	F	2	7	237 ± 5	2	7.90
50	M	2	11	125 ± 3	3	4.17
51	F	3	1	209 ± 8	4	6.97
52	M	3	2	106 ± 5	4	3.53
53	F	3	2	94 ± 11	11	3.13
54	F	3	2	97 ± 7	7	3.23
55	M	3	4	147 ± 6	4	4.90
56	M	3	5	283 ± 5	2	9.43
57	F	3	5	184 ± 7	4	6.13
58	M	3	5	201 ± 3	1	6.70
59	F	4	0	192 ± 3	2	6.40
60	M	4	1	320 ± 8	2	10.67
61	M	4	3	106 ± 4	4	3.53
62	F	4	4	238 ± 5	2	7.93
63	M	4	4	282 ± 8	3	9.40
64	F	4	4	292 ± 6	2	9.73
65	F	4	5	120 ± 3	3	4.00
66	M	4	6	219 ± 11	5	7.30
67	M	4	9	104 ± 2	2	3.47
68	M	5	7	205 ± 2	1	6.83
69	M	5	8	93 ± 3	3	3.10
70	M	5	8	195 ± 8	4	6.50
71	M	5	10	197 ± 3	2	6.57
72	F	6	1	270 ± 6	2	9.00
73	F	6	2	251 ± 5	2	8.37
74	F	6	2	222 ± 5	2	7.40
75	F	6	3	289 ± 10	3	9.63
76	M	6	11	369 ± 15	4	12.30
77	F	8	4	377 ± 10	3	12.57
78	M	8	7	244 ± 6	2	8.13
79	M	10	6	156 ± 5	3	5.20
80	F	12	6	205 ± 3	2	6.83
81	F	14	10	164 ± 3	2	5.47
82	F	15	1	216 ± 12	6	7.20
83	F	15	5	187 ± 2	1	6.23
84	F	15	6	247 ± 10	4	8.23
85	F	17	3	163 ± 5	3	5.43
86	M	17	6	108 ± 6	6	3.60
87	F	17	6	174 ± 6	3	5.80
88	M	19	3	33 ± 5	16	1.10
89	F	19	11	194 ± 2	1	6.47
90	F	20	4	172 ± 7	4	5.73
91	F	20	8	153 ± 6	4	5.10
92	M	21	4	328 ± 11	3	10.93
93	F	24	1	80 ± 3	4	2.67
94	M	25	1	117 ± 3	3	3.90
95	F	25	9	91 ± 2	2	3.03
96	F	27	2	98 ± 5	5	3.27
97	F	27	7	126 ± 6	5	4.20
98	F	27	8	160 ± 4	3	5.33
99	M	28	5	88 ± 8	9	2.93
100	M	28	11	108 ± 6	6	3.60
101	F	29	6	220 ± 3	2	7.33
102	M	29	10	207 ± 8	4	6.90
103	M	30	4	110 ± 4	3	3.67
104	F	31	8	163 ± 3	2	5.43
105	M	32	1	264 ± 5	2	8.80
106	M	32	3	275 ± 7	3	9.17
107	F	32	7	126 ± 4	3	4.20
108	M	33	3	201 ± 8	4	6.70
109	F	33	4	131 ± 3	3	4.37
110	F	33	7	193 ± 6	3	6.43
111	M	33	8	327 ± 9	3	10.90
112	F	36	0	300 ± 8	3	10.00
113	M	36	9	151 ± 7	5	5.03
114	M	36	11	325 ± 5	1	10.83
115	M	39	2	509 ± 14	3	16.97
116	F	41	2	121 ± 3	3	4.03
117	M	42	7	240 ± 3	1	8.00
118	F	42	7	121 ± 2	1	4.03
119	F	43	9	291 ± 7	2	9.70
120	F	45	7	156 ± 6	4	5.20

## 4 Conclusions

In this study, the DIC method was successfully applied to patients with different ages and biotinidase levels, including newborn screening, to adults with a positive correlation to the colorimetric method. Biotinidase deficiency is a treatable disease. Patients treated with an adequate dose of biotin at a young age lead healthy lives. In countries with newborn screening, the main goal is to detect biotinidase in patients when they are healthy. The proposed method enables the diagnosis of patients with biotinidase enzyme deficiency to be made quickly, easily, cheaply, and reliably by processing the color space data of the solutions prepared from serum samples placed in an analyzer box that we designed and produced in the laboratory. Reproducible and reliable results were achieved by (I) using a high-density polyethylene foil material and (II) optimizing the LED light source type, direction, and distance, resulting in homogeneous images free of shadows and reflections from the solutions in the 96-well microplate. The linear working range of PABA was sufficient for serum samples from both patients and healthy subjects. By using the portable analyzer box as an on-site point-of-care test (POC) with a mobile phone camera owned by almost everyone, the biotinidase enzyme activity of patients can be determined anywhere. Moreover, any digital camera can be easily employed for this purpose. Consequently, this paper presents an analytical tool with high potential for determination of biotinidase enzyme activity based on the DIC method.

## Notes

Ethics approval: The study protocol was approved by the Ethical Committee of Cerrahpasa Medical Faculty (No.: E-83045809–604.01.01–576590).
